# ITSxpress: Software to rapidly trim internally transcribed spacer sequences with quality scores for marker gene analysis

**DOI:** 10.12688/f1000research.15704.1

**Published:** 2018-09-06

**Authors:** Adam R. Rivers, Kyle C. Weber, Terrence G. Gardner, Shuang Liu, Shalamar D. Armstrong

**Affiliations:** 1 Genomics and Bioinformatics Research Unit, USDA Agricultural Research Service, Gainesville, FL, 32608, USA; 2 Department of Crop and Soil Sciences, North Carolina State University, Raleigh, NC, 27695, USA; 3 Department of Agronomy, Purdue University, Purdue, IN, 47907, USA

**Keywords:** Amplicon sequencing, marker gene sequencing, internally transcribed spacer, ITS, trimming, QIIME

## Abstract

The internally transcribed spacer (ITS) region between the small subunit ribosomal RNA gene and large subunit ribosomal RNA gene is a widely used phylogenetic marker for fungi and other taxa. The eukaryotic ITS contains the conserved 5.8S rRNA and is divided into the ITS1 and ITS2 hypervariable regions. These regions are variable in length and are amplified using primers complementary to the conserved regions of their flanking genes. Previous work has shown that removing the conserved regions results in more accurate taxonomic classification. An existing software program, ITSx, is capable of trimming FASTA sequences by matching hidden Markov model profiles to the ends of the conserved genes using the software suite HMMER. ITSxpress was developed to extend this technique from marker gene studies using Operational Taxonomic Units (OTU’s) to studies using exact sequence variants; a method used by the software packages Dada2, Deblur, QIIME 2, and Unoise. The sequence variant approach uses the quality scores of each read to identify sequences that are statistically likely to represent real sequences. ITSxpress enables this by processing FASTQ rather than FASTA files. The software also speeds up the trimming of reads by a factor of 14-23 times on a 4-core computer by temporarily clustering highly similar sequences that are common in amplicon data and utilizing optimized parameters for Hmmsearch. ITSxpress is available as a QIIME 2 plugin and a stand-alone application installable from the Python package index, Bioconda, and Github.

## Introduction

The internally transcribed spacer (ITS) between the small subunit (SSU/18S) ribosomal RNA gene and the large subunit (LSU/28S) ribosomal RNA gene is a commonly used phylogenetic marker. The Fungal Barcoding Consortium standardized the practice of ITS sequencing by adopting the region for its efforts ( Schoch *et al.*, 2012), and the major fungal database UNITE uses the region as well ( Kõljalg *et al.*, 2013). It is a common practice to amplify the ITS1 or ITS2 region using primers located in the more conserved 18S/5.8S genes or the 5.8S/28S genes. Previous work has shown that leaving these more conserved regions on the ITS sequence creates miss-assignments. In one study of full length ITS sequences, 11% of the time the ITS1 and ITS2 regions matched one reference sequence but the full sequence including ITS1, ITS2 and the 5.8S did not ( Nilsson *et al.*, 2009). The software package ITSx was developed and subsequently improved ( Bengtsson-Palme *et al.*, 2013; Nilsson *et al.*, 2010) to accurately trim ITS sequences from longer reads. ITSx uses hidden Markov models (HMMs) created for fungi and 17 other groups of eukaryotes to identify the start and stop sites for the ITS region. The software used the HMMER package Hmmscan until version 1.1b when Hmmsearch was substituted for increased speed ( Eddy, 2011).

ITSxpress was created to extend the capabilities of ITSx from marker gene studies using operational taxonomic units (OTUs) to studies using exact sequence variants. Amplicon sequencing creates sequences with errors. In order to distinguish true sequences from sequencing errors, sequences have been clustered into OTU’s by sorting reads by abundance then clustering them in a greedy fashion at a specified percent identity (often 97%). Recently, new methods (e.g. Dada2, Deblur and Unoise) have been published that use statistical models or information theoretic models to identify exact sequence variants that represent true biological sequences ( Amir *et al.*, 2017; Callahan *et al.*, 2016; Caporaso *et al.*, 2010; Edgar, 2016). These methods require the error profiles of individual sequences, which requires trimming each FASTQ sequence ( Cock *et al.*, 2010) to the ITS region of interest. ITSxpress trims FASTQ files for this purpose.

## Methods

### Implementation

ITSxpress rapidly merges and trims paired-end FASTQ sequences to the ITS region of interest for the identification of exact sequence variants. The software merges and error-corrects reads using BBMerge ( Bushnell *et al.*, 2017). The merged FASTQ reads are then sorted by abundance and clustered by default at 99.5% identity to generate a representative set of sequences using VSEARCH ( Rognes *et al.*, 2016). The user may also select dereplication from 98% to 100% identity. These unique sequences are compared to the HMMs used by ITSx version 1.1b ( Bengtsson-Palme *et al.*, 2013) using Hmmsearch ( Eddy, 2011). Read filtering heuristics in Hmmsearch are enabled and reports are filtered as well. The start and stop position of each cluster representative is then used to trim each sequence in the cluster and all original FASTQ sequences that could be merged are returned with the ends trimmed. All major steps (merging, dereplication and Hmmsearch) are multithreaded. The source code is version controlled and tested by continuous integration.

### Operation

ITSxpress is an open source Python package that can be run on Linux or MacOS systems and does not require any special memory or processor configuration. It is available from Github, Pip, Bioconda and as a plugin for QIIME 2. The QIIME 2 package operates on native QIIME 2 .qza files. A typical workflow for an ITS sequencing project would take a set of paired-end FASTQ forward and reverse sequences and return a FASTQ file with merged, trimmed sequences and a log file. Uncompressed FASTQ or Gzip compressed FASTQ files can be used. The command line version of ITSxpress accepts interleaved, paired-end files, forward and reverse paired end files, and single-ended files. The QIIME 2 plugin version of ITSxpress accepts a .qza QIIME 2 artifact file of the type “PairedEndSequencesWithQuality” or “SequencesWithQuality” that contains one or more samples with single or paired data. It merges (if paired) and trims all samples and returns a QIIME 2 artifact file containing single-ended sequences with quality or paired-end sequences with quality that can be used for sequence variant calling by DADA2 or Deblur ( Amir *et al.*, 2017; Callahan *et al.*, 2016).

### Testing

To compare the speed and trimming results of ITSxpress, we compared the ITS1 and ITS2 sequences from 15 soil samples collected from the rhizosphere of maize in fields with different winter cover crops. ITS1 reads were amplified using the ITS1F/ITS2 primer set ( Gardes & Bruns, 1993; White *et al*., 1990). ITS2 reads were amplified using the ITS3/ITS4 primer set ( White *et al*., 1990). Reads were multiplexed and sequenced on an Illumina Miseq in 2x300bp run mode using version 3.0 chemistry.

**Figure 1.**
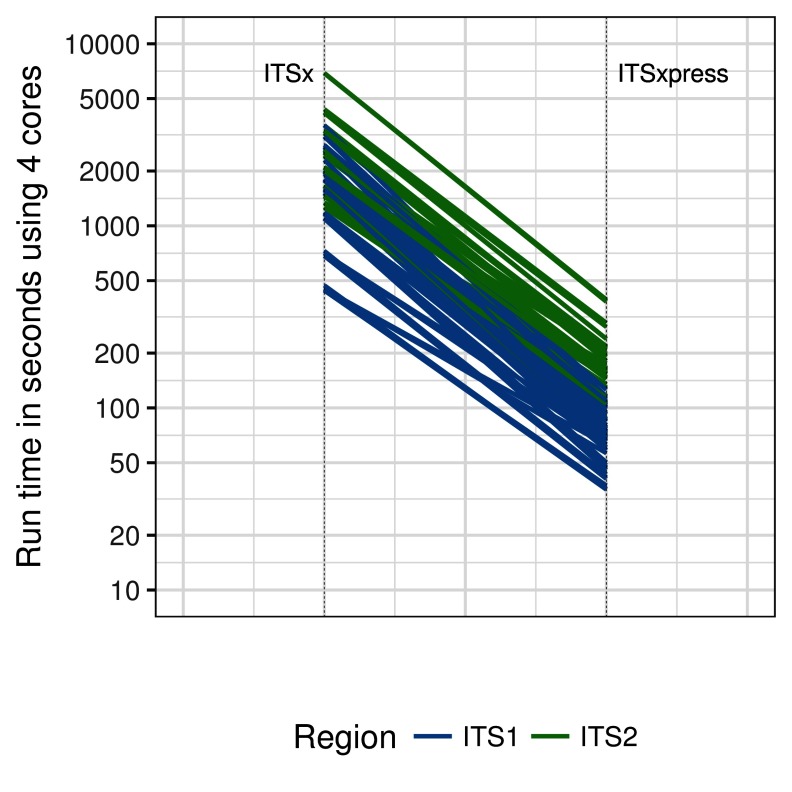
The run times for ITS1 and ITS2 samples processed using ITSx and ITSxpress using 4 logical compute cores. N=5 for each of the 30 samples.

**Figure 2.**
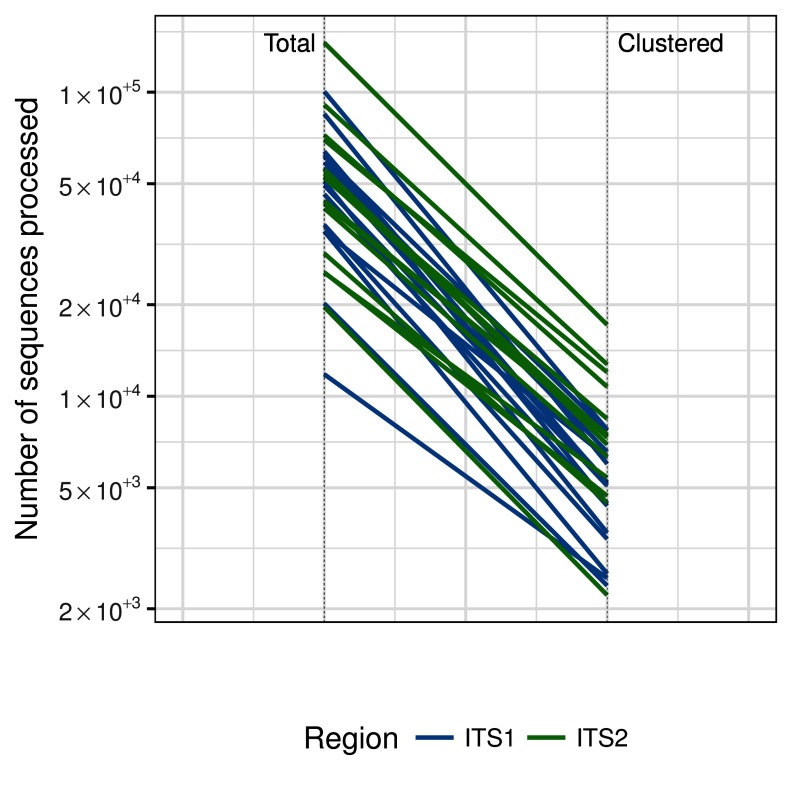
The number of total reads and the number or representative reads after clustering at 99.5% identity.

Tests of ITSxpress and ITSx performance were run on single compute nodes with 2 x 10 core Intel Xeon Processors (E5-2670 v2 2.50GHz 25MB cache) with hyper-threading enabled, 128GB DDR3 ECC memory and two Intel DC S3500 Series SATA 6.0Gb/s SSDs. For the first test of trimming speed, 5 replicates were run where 15 ITS1 and 15 ITS2 samples were trimmed using ITSxpress and ITSx with 4 logical cores. Trimming was done using ITSxpress with default settings. ITSx was run with multithreading and heuristic filtering turned on and only the fungal database selected. The running times for ITSx and ITSxpress were plotted on a log scale, Figure 1. The number of total reads in each sample and reads remaining after clustering at 99.5% identity are shown on a log scale, Figure 2.

**Figure 3.**
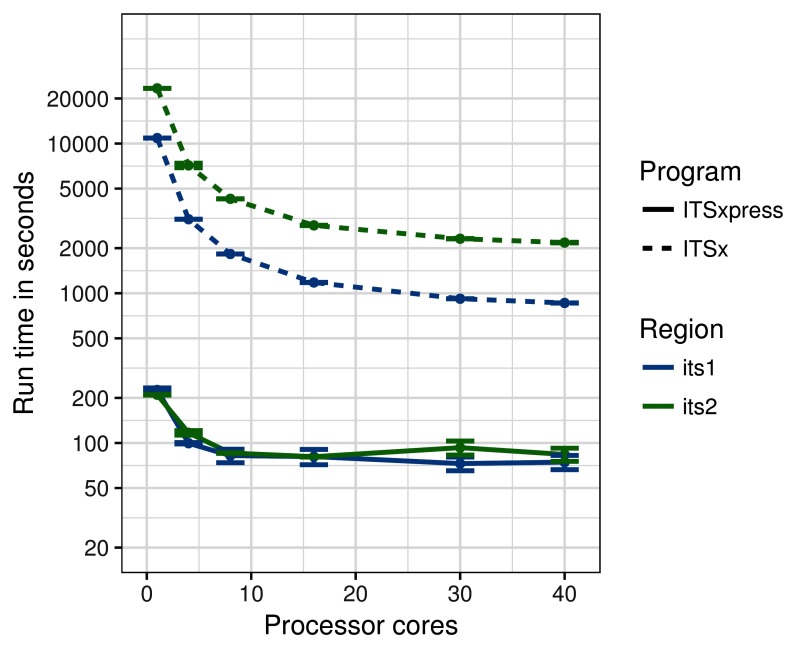
The mean and standard error of run times for of ITSx and ITSxpress on multiple logical cores. The largest samples from the ITS1 (n=100,543) and ITS2 (n=145,499) datasets were selected for analysis. N=5 for each core/sample combination.

To compare the performance of ITSxpress and ITSx as computer cores were added, tests were run on the ITS1 and ITS2 sample with the largest numbers of sequences (ITS1: n=100543 16% unique, ITS2: n=145499, 30% unique). The sample was processed 5 times with 1, 4, 8, 16, 30, and 40 virtual compute cores. The mean and standard error were plotted, Figure 3. Program settings were the same as in the first test.

The trimming positions from ITSx and ITSxpress were compared for every ITS1 and ITS2 sequence. If a read was not trimmed identically by ITSx and ITSxpress, it was globally aligned and the start and stop positions were compared. Alignment was done using the Biopython Pairwise2 implementation of a global alignment function with the parameters (match score: 2, mismatch penalty: -1, gap opening penalty: -0.5, gap extension penalty: -0.1) ( Cock *et al.*, 2009).

## Results

When using 4 cores, ITSxpress trimmed ITS1 region samples a median of 23 times faster (Bayesian 95% Highest Density Interval (HDI) 7 – 32) than ITSx, HDI interval calculated with the R package HDInterval ( Meredith & Kruschke, 2018). ITSxpress trimmed the ITS2 region 14 times faster (95% HDI interval 8 – 24) than ITSx ( Figure 1). Clustering at 99.5% identity reduced the number of reads used for Hmmsearch by a median of 71 times (95% HDI 17 – 95) for ITS1 and 36 times (95% HDI 21 – 52) for ITS2, Figure 2.

Global alignment was used to compare the trimming results of ITSx and ITSxpress for reads that were not identical. When reads were clustered at 99.5% identity, the default behavior, ITSxpress and ITSx trimmed 99.822% (n=773021) of reads in the ITS1 region within 2 bases of each other and 99.099% (n= 782385) of reads in the ITS2 region within 2 bases of each other. When reads were dereplicated at 100% identity ITSxpress and ITSx trimmed 99.992% (n= 773019) of reads in the ITS1 region within 2 bases of each other and 99.864% (n=782582) of reads in the ITS2 region within 2 bases of each other.

## Discussion

ITSxpress increases the trimming speed of ITS sequences by clustering reads and optimizing the parameters for Hmmsearch. Most of the decrease in running time is attributable to clustering. Clustering at 99.5% identity resulted in reducing the number of sequences by a median of 71 to 36 times for the ITS 1 and ITS2 regions. The time complexity for Hmmsearch on a single core is approximately linear with the number of sequences so decreases in the number of sequences significantly decrease running time. The time required for clustering varies, for dereplication at 100% identity VSEARCH uses the rapid CityHash64 function ( Rognes *et al.*, 2016). For clustering at less than 100% reads are sorted by abundance then clustered using greedy search. These steps take time but are faster than Hmmsearch and scale sub-linearly, resulting in median speed increases of 6-9x for the sequences dereplicated at 100% identity and 14-23x for the sequences clustered at 99.5% identity.

Both ITSx and ITSxpress use Hmmsearch, the same hmm models, and run using multiple cores. ITSxpress uses empirically tuned Hmmsearch heuristic values of 1×10 ^-6^ for F1, F2 and F3 which show increased speed and little loss of sensitivity. ITSx uses Hmmsearch’s default values of 1×10 ^-2^ for F1, 1×10 ^-3^ for F2 and 1×10 ^-5^ for F3 when the “--heuristics” flag is set.

ITSx and ITSxpress scale differently as cores are added. ITSxpress spends about half its time clustering when the clustering identity is below 100%, and for a typical ITS sample this reduces the number of sequences to be analyzed by Hmmsearch to the point where parallelizing Hmmsearch does not result in large speed gains. This trait is beneficial for users using laptop or desktop computers because they can trim a typical ITS sample in less than a minute using 1–4 cores. Both programs use Hmmsearch for the most computationally intensive part of their workflows. ITSx benefits from Hmmsearch parallelization up to about 10 cores but then the increases decline; the nonlinear scaling of Hmmsearch is noted in the HMMER User Guide. ( Eddy, 2011).

ITSx and ITSxpress trim most sequences exactly the same. At 100% identity one in 12,500 ITS1 sequences and one in 735 ITS2 sequences differ by more than two bases. This may be caused by differences in the heuristic settings for Hmmsearch. At 99.5% identity clustering the differences are greater, with one in 560 ITS1 sequences and one in 110 ITS2 sequences differing by more than two bases. At 99.5% identity, sequences from 600-800bp can be 3bp different, but be clustered together. Substitutions do not affect the trimming position, but insertions or deletions do, accounting for some of the difference. The clustering identity can be set to as low as 98% identity to accommodate special uses but lowering the identity below 99.5% is not generally recommended since ITSxpress is quite fast even at 100% identity.

ITSxpress quickly merges reads and trims the selected ITS region from a range of amplicon samples. It trims FASTQ files allowing for the use of newer sequence variant methods of exact sequence clustering and is available as a command line application and as a plugin for QIIME 2.

## Software and data availability

### Software

The source code for the stand-alone version of ITSxpress version 1.6.1 used for this manuscript is available from: https://doi.org/10.5281/zenodo.1317575 ( Rivers, 2018a). This software is available under the terms of the Creative Commons Zero "No rights reserved" data waiver (CC0 1.0 Public domain dedication).

Updated versions of the ITSxpress software are available from:

- Github: https://github.com/USDA-ARS-GBRU/itsxpress
- The Python Package index: https://pypi.org/project/itsxpress/
- Bioconda: https://bioconda.github.io/recipes/itsxpress/README.html

The QIIME 2 plugin for ITSxpress is available from: http://doi.org/10.5281/zenodo.1317579 ( Weber & Rivers, 2018); Github ( https://github.com/USDA-ARS-GBRU/q2_itsxpress); and the Python Package index ( https://pypi.org/project/q2_itsxpress/). This software is available under the terms of the CC0 1.0 Public domain dedication.

The computer code used to benchmark the software and generate the figures in this paper is available at: http://doi.org/10.5281/zenodo.1317585 ( Rivers, 2018b); and Github ( https://github.com/USDA-ARS-GBRU/itsxpress-paper). The code is also available under the terms of the CC0 1.0 Public domain dedication.

### Data

Data used in this study are deposited in the NCBI Sequence Read Archive under the accessions listed in NCBI BioProject Accession PRJNA483055.
