# Supplementary material for: ITSxpress: Software to rapidly trim internally transcribed spacer sequences with quality scores for marker gene analysis
Source: F1000Res. 2018 Sep 6;7:1418. [Version 1] doi: 10.12688/f1000research.15704.1 (PMC6206612; doi:10.12688/f1000research.15704.1)
Supplement: Peer review history

# Peer review: Referee response for version 1

**DOI:** 10.5256/f1000research.17138.r38810

**Published:** 2018-10-24

**Authors:** Johanna B. Holm

**Affiliations:** 1 Institute for Genome Sciences, Department of Microbiology and Immunology, University of Maryland School of Medicine, Baltimore, MD, USA

This report introduces an updated version of ITSx making the tool applicable to exact sequence variant analyses as opposed to operational taxonomic unit analyses. The tool is meant to be implemented upstream of sequence variant calling algorithms, producing dereplicated and merged reads. These reads can then be used in the deblur or dada2 algorithms for calling the exact sequence variants. 

 Minor Issues: 

- Methods/Operation: Because there is clear instruction for use of the ITSxpress product in qiime2, it would be helpful to add a sentence regarding the use of the product in the dada2 workflow, as most users are accustomed to running the forward and reverse reads through the dada2 algorithm separately, and merging afterwards. Would the correct methodology be to run dada2(ITSxpress file) followed by sequence table production (skipping the merge step)? 
- Methods/Implementation: replace "sorted by abundance and clustered" with "sorted by abundance and dereplicated", for clarity. 

I have read this submission. I believe that I have an appropriate level of expertise to confirm that it is of an acceptable scientific standard.

# Peer review: Referee response for version 1

**DOI:** 10.5256/f1000research.17138.r38059

**Published:** 2018-09-19

**Authors:** J. Gregory Caporaso

**Affiliations:** 1 The Pathogen and Microbiome Institute, Department of Biological Sciences, Northern Arizona University, Flagstaff, AZ, USA

The authors present ITSxpress, an approach for trimming flanking rRNA regions from ITS sequence data. This is effectively a replacement for ITSx, which performed this process on fasta files after sequence clustering. By working with fastq data, ITSxpress allows this trimming to be applied to fastq files, which in turn allows for the application of modern amplicon analysis workflows. 

Major points:

*The user may also select dereplication from 98% to 100% identity.*

 The above sentence should probably be replaced with the following, since dereplication usually refers to clustering at 100% identity: *The user may also choose to cluster at between 98% and 100% identity.*

*The start and stop position of each cluster representative is then used to trim each sequence in the cluster*

 How does this work if there were insertions and/or deletions between the cluster representative sequence and each cluster member? Wouldn't the position numbers be incorrect in that case? 

 How does a lower percent identity threshold for clustering impact accuracy (as compared to ITSx) and runtime? I'm wondering if it's worth it, for example, to run the clustering step at 98%, or maybe even lower, for quicker run time. Exploring accuracy and runtime as functions of percent identity seems like a missing piece of this study since in some cases the runtimes can be fairly long (e.g., 80 minutes) for a pre-processing step. 

Minor points:

*miss-assignments* should be *mis-assignments*

**

*OTU’s *is used in several places where *OTUs* should be used

*It merges (if paired) and trims all samples*

 Should that say "trims all sequences"?

I have read this submission. I believe that I have an appropriate level of expertise to confirm that it is of an acceptable scientific standard.
